# Implication of *TrkC‐miR2* in neurotrophin signalling pathway regulation through *NGFR* transcript targeting

**DOI:** 10.1111/jcmm.16415

**Published:** 2021-03-06

**Authors:** Sadat Dokaneheifard, Bahram M. Soltani

**Affiliations:** ^1^ Department of Molecular Genetics Faculty of Biological Sciences Tarbiat Modares University Tehran Iran; ^2^ Medical Biology Research Center Kermanshah University of Medical Sciences Kermanshah Iran

**Keywords:** neurotrophin signalling pathway, *NGFR*, *TrkC‐miR2*

## Abstract

TrkC and NGFR neurotrophin receptors are associated with cell death, cancer and differentiation. *TrkC‐miR2,* which is located in *TrkC* gene, is known to regulate Wnt signalling pathway, and its influence on other signalling pathways is under investigation. Here, through RT‐qPCR, dual‐luciferase assay and Western blotting we reveal that *TrkC‐miR2* targets *NGFR*. Overexpression of *TrkC‐miR2* also affected *TrkA*, *TrkC, NFKB*, *BCL2* and *Akt2* expressions involved in neurotrophin signalling pathway, and elevated survival rate of HEK293t and U87 cells was distinguished by flow cytometry and MTT assay. Consistently, an opposite expression correlation was obtained between *TrkC‐miR2* and *NGFR* or *TrkC* for the duration of NT2 differentiation. Meanwhile, *TrkC‐miR2* down‐regulation attenuated NT2 differentiation into neural‐like cells. Overall, here we present in silico and experimental evidence showing *TrkC‐miR2* as a new controller in regulation of neurotrophin signalling pathway.

## 1 BACKGROUND

Neurotrophins, secretory growth factors, encourage existence, growth, development and function of neurons and other cell types via binding to multiple specific receptors including TrkA, TrkB, TrkC and NGFR.^1^ In some cell types, expression of Trk family receptors either stimulates cell proliferation or differentiation of neural cells.^2^ *TrkC* acts as an oncogene or a tumour suppressor and is associated with controlling of growth and survival of various cancer cells in humans.^3^, ^4^, ^5^ It is well known that TrkC signalling stimulates PI3K, Akt and MAPK pathways, where their mechanisms are in deep investigation.^1^ p75^NGFR^ or NGFR acts either as an oncogene or a tumour suppressor depending on the content of the cells. NGFR’s function continued conflicting in some cancers, probably due to the heterogeneity.^6^ No intrinsic enzymatic activity has been reported for NGFR; however, its signal transduction depends on the recruitment of other cell surface receptors such as TrkC. Consequently, there is a diversity of consequences in NGFR signalling depending on the interplay between neurotrophin cell surface receptors. For example, NGFR is highly expressed in melanoma and thyroid carcinoma, whereas it is down‐regulated in stomach, bladder, liver and prostate cancers.^6^ MicroRNAs or miRNAs are endogenous extremely conserved RNAs with ~ 21 nucleotides in length produced in many organisms and implicated in regulation of several crucial cell processes including cell death, survival and differentiation and also in many diseases.^7^ They may interplay between different receptors of the signalling pathways. We have previously introduced *hsa‐miR‐6165* located in *NGFR* gene intron,^8^ with functionality during the course of NT2 cell differentiation.^9^ We have also introduced *TrkC‐miR2* at the vicinity of *hsa‐miR‐11181* regulating Wnt pathway.^10^, ^11^ Here, we hypothesized interplay between neurotrophin receptors through the *TrkC‐miR2* and confirmed the effect of *TrkC‐miR2* on NGFR gene transcript.

## 2 METHODS

### 2.1 In silico analysis for prediction of *TrkC‐miR2* candidate target genes

In order to predict *TrkC‐miR2* potential target genes, we used DIANA‐microT and RNAHybrid online tools. DAVID, Geneset2 and Diana‐miRPath online software packages were applied to find the pathways are affected by *TrkC‐miR2*.^12^

### 2.2 Cell lines and tissue samples

RPMI 1640 was used as the media for culturing HeLa, HepG2, U87MG, 1321N1, Daoy, A172 and SK‐N‐MC cell lines. SW480, HEK293 t and NT2 cells were maintained in DMEM‐HG. These media were supplemented with 10% foetal bovine serum (FBS).^10^, ^12^ Tissue samples were freshly obtained from Imam Hospital, in Tehran, Iran, and then stored in −80 until used.

### 2.3 DNA constructs

Human genomic DNA extraction,^13^ *TrkC‐miR2* precursor cloning using the primers named Int‐F and Int‐R both in sense and in antisense directions, cloning of the scrambled sequence as a control construct,^14^ and the strategy used for *NGFR* 3'UTR cloning ^10^, ^11^, ^12^, ^15^ have been previously reported. In order to clone the region corresponding to *TrkC‐premir2* sense and antisense sequences, about 802bp of human *TrkC*‐intron‐14 was PCR‐amplified using Int‐F and Int‐R primers (Table 1) and cloned into pEGFP‐C1 expression vector (Clontech) downstream of GFP sequence, both in sense and in antisense directions.^11^

**TABLE 1:** The list of used oligo sequences

Primer name	Primer sequence, 5’ to 3’	Amplicon’ size (base pairs)
*TrkC*‐real time	Forward: CCTGTGTCCTGTTGGTGGTTCTC Reverse: GAGTCATGCCAATGACCACAGTGTC	195
*TrkC*‐miR2‐5p	GGCTGGGGATTCTGAGCT	
U48	Forward: TGACCCCAGGTAACTCTGAGTGTGT	
Anchored OligodT‐CT	GCGTCGACTAGTACAACTCAAGGTTCTTCCAGTCACGACG(T)18AG	
Anchored OligodT‐GC	GCGTCGACTAGTACAACTCAAGGTTCTTCCAGTCACGACG(T)18GC	
Universal‐inner	AACTCAAGGTTCTTCCAGTCACG	
*NGFR*‐real time	Forward: CCGAGGCACCACCGACAACC Reverse: GGGCGTCTGGTTCACTGGCC	151
*GAPDH*	Forward: GCCACATCGCTCAGACAC Reverse: GGCAACAATATCCACTTTACCAG	115
*TrkC*‐Intron	Int‐F : CTGGCGGCCGCTGAACAAGGGAGATGGCTCAGTGG Int‐R: TAGACGCGTGGCTTTGCTGTCACCGCTGAGG	802
*NGFR*‐3’UTR	Forward:CCCCTCGAGCCACATTCCGACAACCGATGC Reverse:GCCCAAGAAATGATTACACAGGAGG	1920

### 2.4 RNA extraction

TRIzol kit (Invitrogen) was used for total RNA extraction according to the protocol reported by its manufacturer and then was treated with DNase I purchased from Fermentas.

### 2.5 RNA polyA adenylation, cDNA synthesis and RT‐qPCR

In order to examine *TrkC‐miR2* expression level, polyA tail was initially added to the extracted total RNA by using polyA polymerase enzyme (NEB), and cDNA was then synthesized by using two anchored oligodT (Table 1) primers against both TrkC‐miR2 isomiRs according to the previously reported protocol.^8^, ^10^, ^11^ *TrkC‐miR2* has two isomiRs, which are different in 2 last nucleotides at their 3’‐ends. These two isomiRs were identified in our previous study.^11^ The sequences of *TrkC‐miR2‐5p‐GC* and *TrkC‐miR2‐5p‐CT* are GGCTGGGGATTCTGAGCTGC and GGCTGGGGATTCTGAGCT, respectively. 1 μL of cDNA products was then applied for RT‐qPCR.^8^, ^11^ *U48* and *GAPDH* were used as the control genes. Expression data normalization was performed using 2^‐ΔCt^ and 2^‐ΔΔCt^ method.^16^

### 2.6 Dual‐luciferase assay

A Dual‐Luciferase Reporter Assay System (Promega) was utilized for measurement of luciferase activity two days post‐HEK293‐T cell transfection. The controls and experimental conditions for performing this experiment have been reported previously.^10^, ^11^, ^15^

### 2.7 Primer designing

Primer sequences that were designed using IDT, NCBI Primer‐blast and MWG tools are listed in table 1.

### 2.8 NT2 cell differentiation

In order to differentiate NT2 cells into neural‐like cells, retinoic acid (RA) treatment was applied, according to the previously reported procedure.^17^ Also, NT2 cells were transfected with the vector overexpressing anti‐*TrkC‐premir2*, 10 days after beginning differentiation. Expression alteration of *Oct4*, *PAX6*, *hsa‐miR‐302* and *hsa‐miR‐145* differentiation markers along with morphological changes was used for following up the successful differentiation process.

### 2.9 Overexpression of *TrkC‐premir2*

A pEGFP‐C1 vector containing and expressing *TrkC‐miR2* precursor was surrounded in Lipofectamine 2000 purchased from Invitrogen, and utilized for transfection of the studied cell lines. Successful transfection was then ensured via GFP microscopy one day post‐transfection.

### 2.10 Western blotting

After loading of 30μg of each protein on to 12% SDS‐PAGE, protein transferring was performed into PVDF membrane. 5% skim milk was used for membrane blocking for 1h at room temperature. The primary antibody incubation was done for 12h at 4°C and then followed by secondary antibody incubation for 1h at RT. Amersham ECL Prime Western Blotting Detection Reagent Kit was used for visualization of blot. Western blotting data were quantitated using the TotalLab Quant software.

### 2.11 Cell Cycle Analysis

The protocol performed for cell cycle analysis has been described in our previous paper.^11^

### 2.12 Statistical Analysis

Statistical study was completed by GraphPad Prism 5.04. In order to analyse the apoptosis experiment statistically, the Bonferroni test was employed following the repeated‐measures ANOVA test.

## 3 RESULTS

### 3.1 Regulation of neurotrophin signalling pathway by *TrkC‐miR2*

**FIGURE 1:**
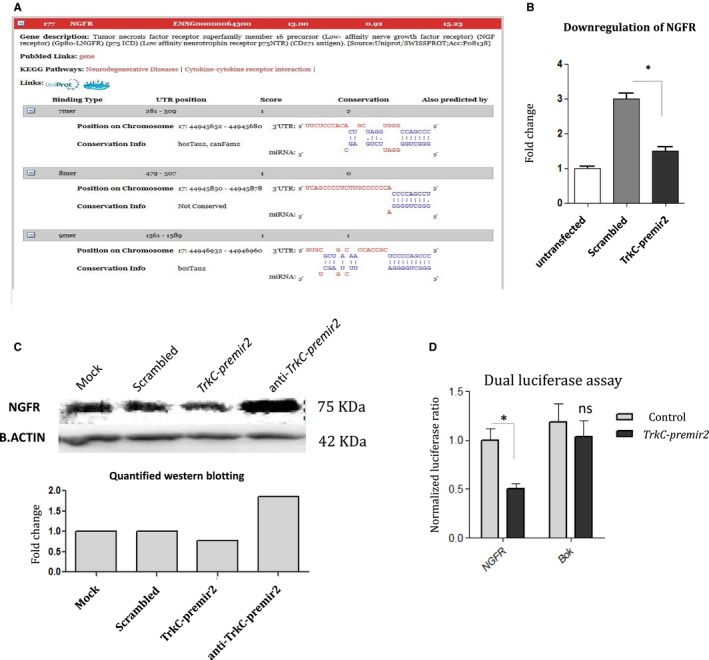
Direct interaction between *TrkC‐miR2* and *NGFR*. A, The alignment and conservation status of three of MREs predicted by Dianna lab software for *TrkC‐miR2* with *NGFR*. B, RT‐qPCR indicates *NGFR* down‐regulation following *TrkC‐premir2* overexpression within SW480 cells, compared with the related negative controls. C, Western blotting gel image shows NGFR protein level reduction following the *TrkC‐premir2* overexpression in HUH7 cells. The cells were either transfected with the vector encoding *TrkC‐premir2* or the related controls. Results show down‐regulation of NGFR protein as a result of *TrkC‐premir2* overexpression. D, Analysis of dual‐luciferase assay revealed supporting evidence in direct interaction between *NGFR* and *TrkC‐miR2*. In this assay, 3’UTR of *Bok* gene was used as an off‐target control. Results are the mean of triplicate; *P* <.05

Using Dianna lab software, about 700 target genes were predicted for *TrkC‐miR2*. There are three poorly conserved MREs within the 3’UTR sequence of *NGFR* (ENSG00000064300) gene (Figure 1A). Following the RT‐qPCR assessment of *NGFR* endogenous expression level within SW480 cells (data not shown), *TrkC‐premiR2* was overexpressed in these cells, which resulted in 50% down‐regulation of *NGFR* expression (Figure 1B). Furthermore, Western blotting verified about 8% decrease in NGFR protein level following the *TrkC‐premir2* overexpression in comparison with the cells transfected by scrambled construct. Consistently, overexpression of a vector containing an antisense sequence against *TrkC‐premir2* resulted in NGFR protein level elevation (Figure 1C). When NGFR 3'UTR was cloned in a vector at downstream of luciferase ORF, and coexpressed with *TrkC‐premir2*, dual‐luciferase assay showed about 50% reduction in luciferase counts supporting a direct interaction between *TrkC‐miR2* and 3'UTR of *NGFR* (Figure 1D).

### 3.2 *TrkC‐premir2* overexpression effect on the expression of the genes implicated in neurotrophin signalling pathway

**FIGURE 2:**
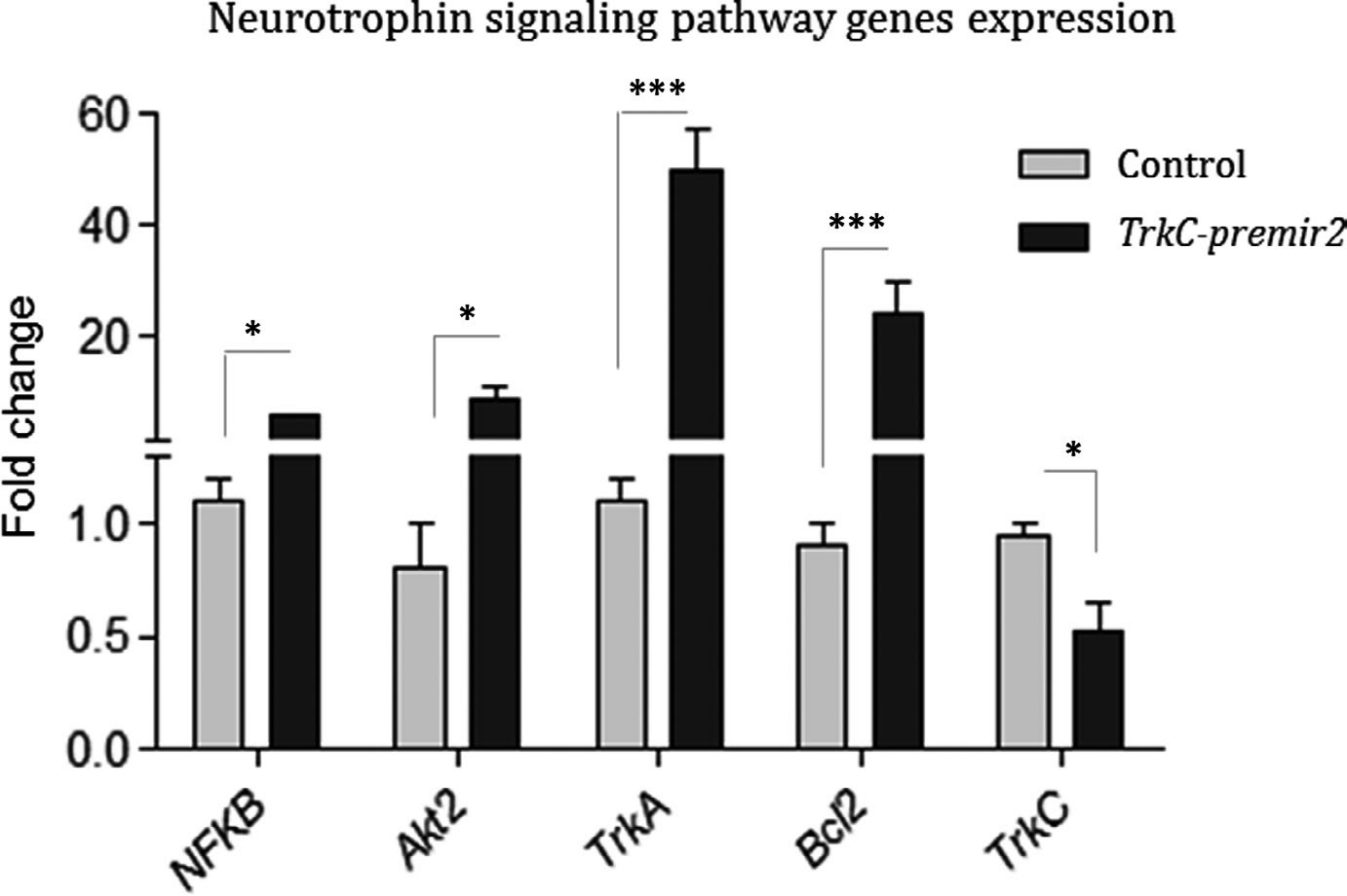
Consequence of *TrkC‐premir2* overexpression on the expression level of the downstream genes in neurotrophin signalling pathway. Elevated expression of the genes implicated in subpathways of neurotrophin signalling pathway following *TrkC‐premir2* overexpression in comparison with the cells transfected with empty vector as a negative control. Except *TrkC*, most of tested genes were up‐regulated following overexpression of *TrkC‐premir2*. SD of duplicate experiments is shown by the error bars. *GAPDH* was applied as an internal control. Expression data were normalized using 2^‐ΔΔCt^ method

The global consequence of *TrkC‐premir2* overexpression effect on downstream genes of neurotrophin signalling in U87 cell line was additionally examined using RT‐qPCR. Results indicated that the expression levels of *TrkA*, *Akt2*, *NF‐κB* and *BCL2* genes have been highly elevated following the *TrkC‐premir2* overexpression, compared with the mock control. Nevertheless, *TrkC* gene expression level has been reduced within the same cells (Figure 2).

### 3.3 Detection of endogenous *TrkC‐miR2* in human cell lines and brain tumour specimens

**FIGURE 3:**
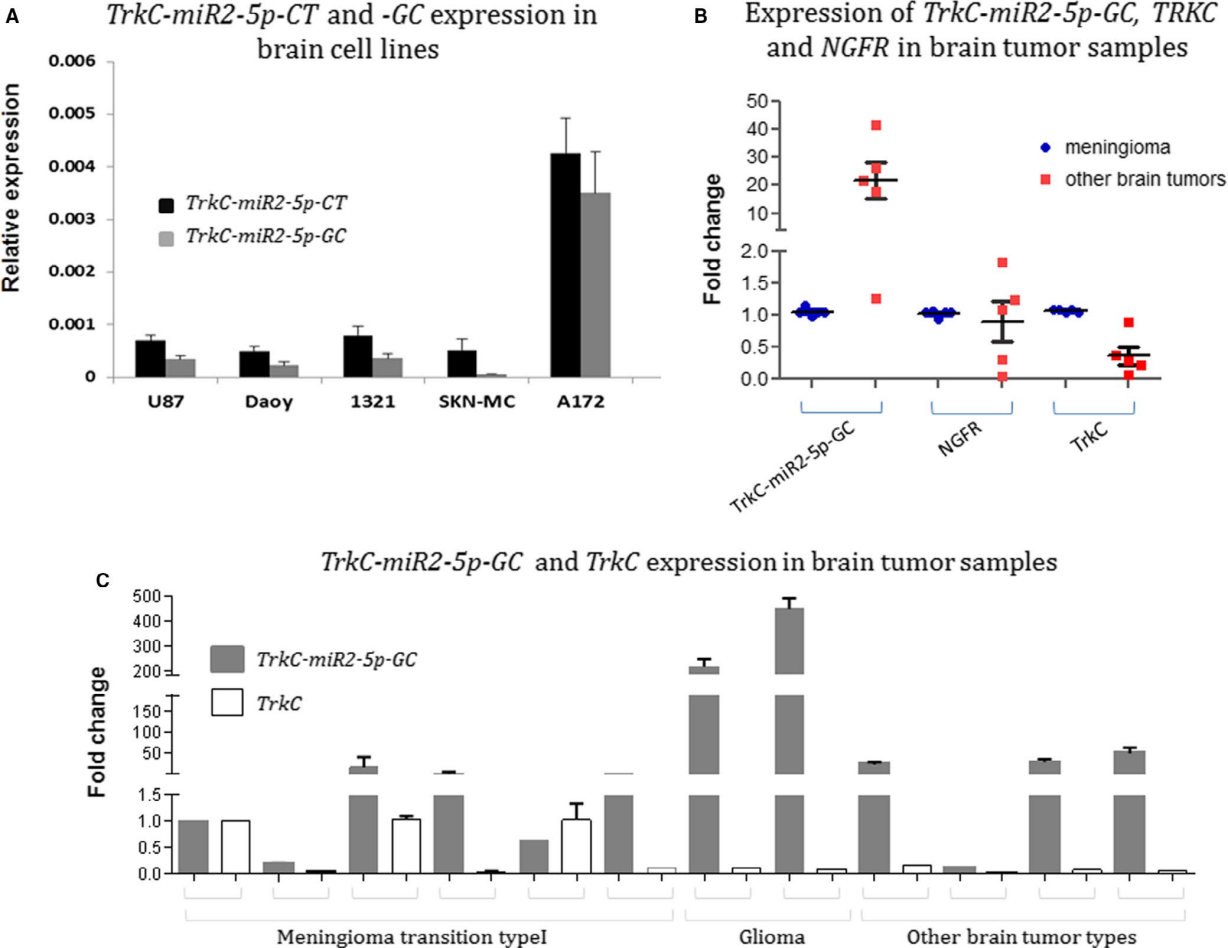
*TrkC*, *NGFR* and *TrkC‐miR2‐5p* expression status in brain tumours and cell lines. A, Detection of *TrkC‐miR2‐5p* isomiRs in brain (glioma and non‐glioma) cell lines. In glioma‐originated A172 cells, *TrkC‐miR2‐5p* isomiR’s expression level was significantly higher than other cell lines. B, *TrkC‐miR2‐5p‐GC* is significantly elevated in brain tumours (about 20 folds) compared with the meningioma transition type 1 tissue samples (*P* <.001) analysed by the Mann‐Whitney method. However, the expression of *TrkC* and *NGFR* was decreased in related samples. C, The figure shows *TrkC‐miR2‐5p‐GC* and *TrkC* expression levels in different brain tumour tissue specimens. RT‐qPCR was performed for the analysis of *TrkC‐miR2‐5p‐GC* expression in the tumour specimens in comparison with their expression level in the meningioma transition type I. Here, the maximum expression level of *TrkC‐miR2‐5p‐GC* was distinguished in glioma samples. *GAPDH* and *U48* were applied as internal controls for normalization of protein coding and miRNA expressions, respectively

Status of endogenous expression level of *TrkC‐miR2* was identified through RT‐qPCR in astrocytoma (1321N1), glioblastoma (A172 and U87MG), medulloblastoma (Daoy) and neuroblastoma (SK‐N‐MC) human brain tumour cell lines (Figure 3A). The highest expression level of *TrkC‐miR2* was identified in A172. The endogenous *TrkC‐miR2‐5p‐GC* isomiR was also detected in primary brain tumour specimens (Figure 3B,C). Although, *TrkC‐miR2*‐5p‐GC was relatively expressed at low level in most of the examined brain cancer biopsies, the highest expression level of it was detected in glioma biopsies (Figure 3B,C) compared with meningioma transition type 1 tissue samples as the control. On the other hand, both *TrkC* (significant) and *NGFR* (non‐significant) genes were down‐regulated in the examined tumour samples (Figure 3B). Interestingly, it seemed that *TrkC‐miR2‐5p‐GC* is expressed independent of *TrkC* (as the *TrkC‐miR2* host gene) in all of the tested tumour samples (Figure 3C).

### 3.4 Anti‐apoptotic effect of *TrkC‐premir2* in cell lines

**FIGURE 4:**
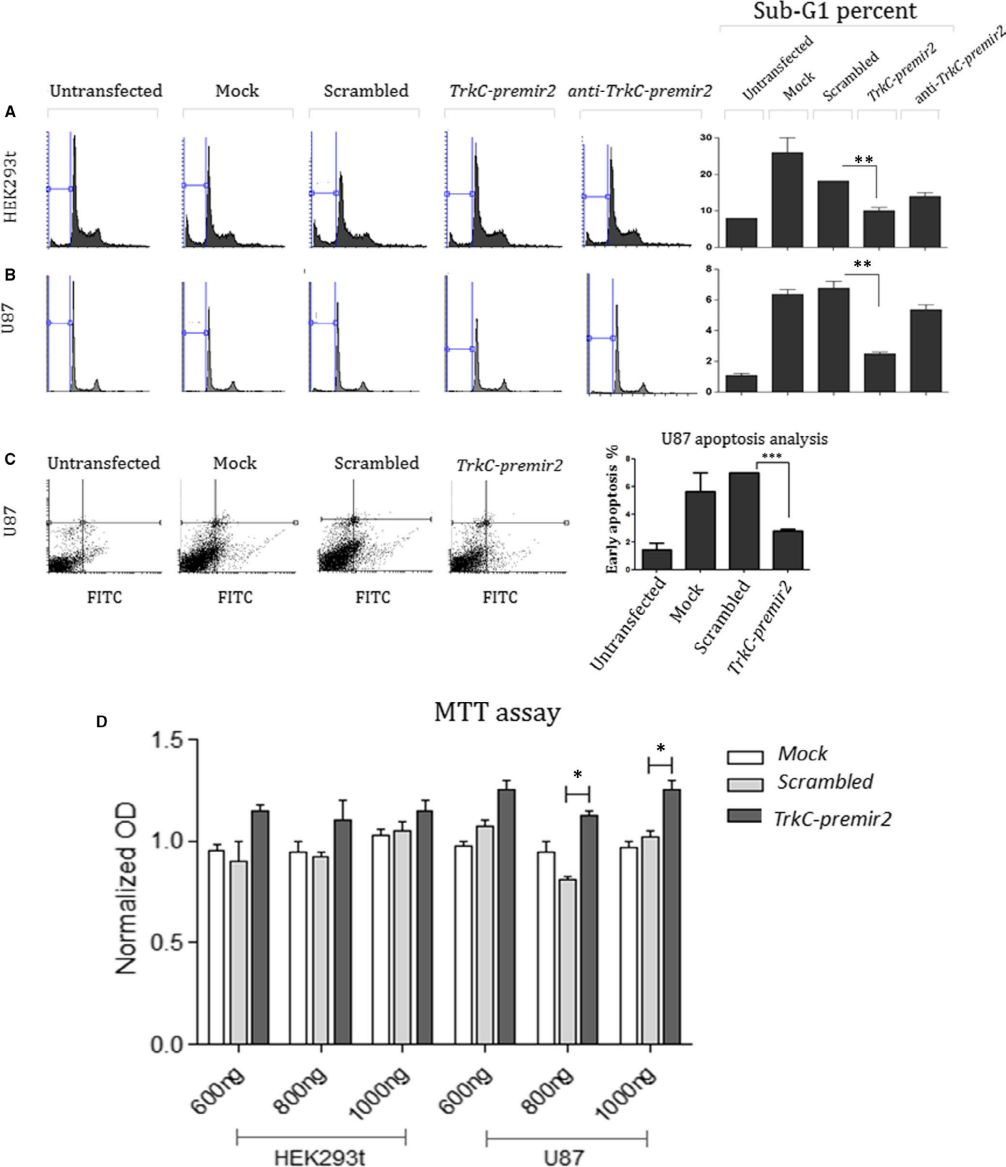
Implication of *TrkC‐premir2* overexpression in the cell cycle status alteration of the cells. A and B, PI staining of the HEK293 t and U87 cells after the overexpression and knock down of *TrkC‐premir2*. Significant decrease in the distribution of sub‐G1 cell population was documented in HEK293 t (A) and U87 (B) cells following overexpression of *TrkC‐premir2*. Consistently, knockdown of *TrkC‐premir2* in the same cells had a reverse effect on sub‐G1 percentage. C, The figure displays annexin‐PI analysis of transfected U87 cells. The gate setting revealed that most of the cells that overexpress *TrkC‐premir2* have less distribution in early apoptosis stage (bottom right), compared with the negative controls. D, The figure illustrates the outcome of MTT test in the HEK293 t and U87 cells containing a vector encoding *TrkC‐premir2*. Survival rate of the transfected U87 and HEK293 t cells was meaningfully raised following *TrkC‐premir2* overexpression. SD of triplicate experiments is shown by error bar

In order to look at the outcome of *TrkC‐premir2* overexpression on the cell cycle status, U87 and HEK293 t cell lines were transfected by a vector overexpressing *TrkC‐premir2*. A significant decrease in sub‐G1 cell population was observed following *TrkC‐premir2* overexpression in HEK293 t and U87, in comparison with the cells containing the negative control vector. Inversely, knockdown of this miRNA within the above‐mentioned cell lines attenuated its cell survival effect (Figure 4A,B). An anti‐apoptotic influence of *TrkC‐premir2* overexpression in U87 was also confirmed by using annexin V test (Figure 4C). Further, MTT assay results confirmed survival effect of *TrkC‐premir2* overexpression in U87 and HEK293 t cells (Figure 4D).

### 3.5 *TrkC‐miR2* expression alteration for the duration of NT2 cell differentiation

**FIGURE 5:**
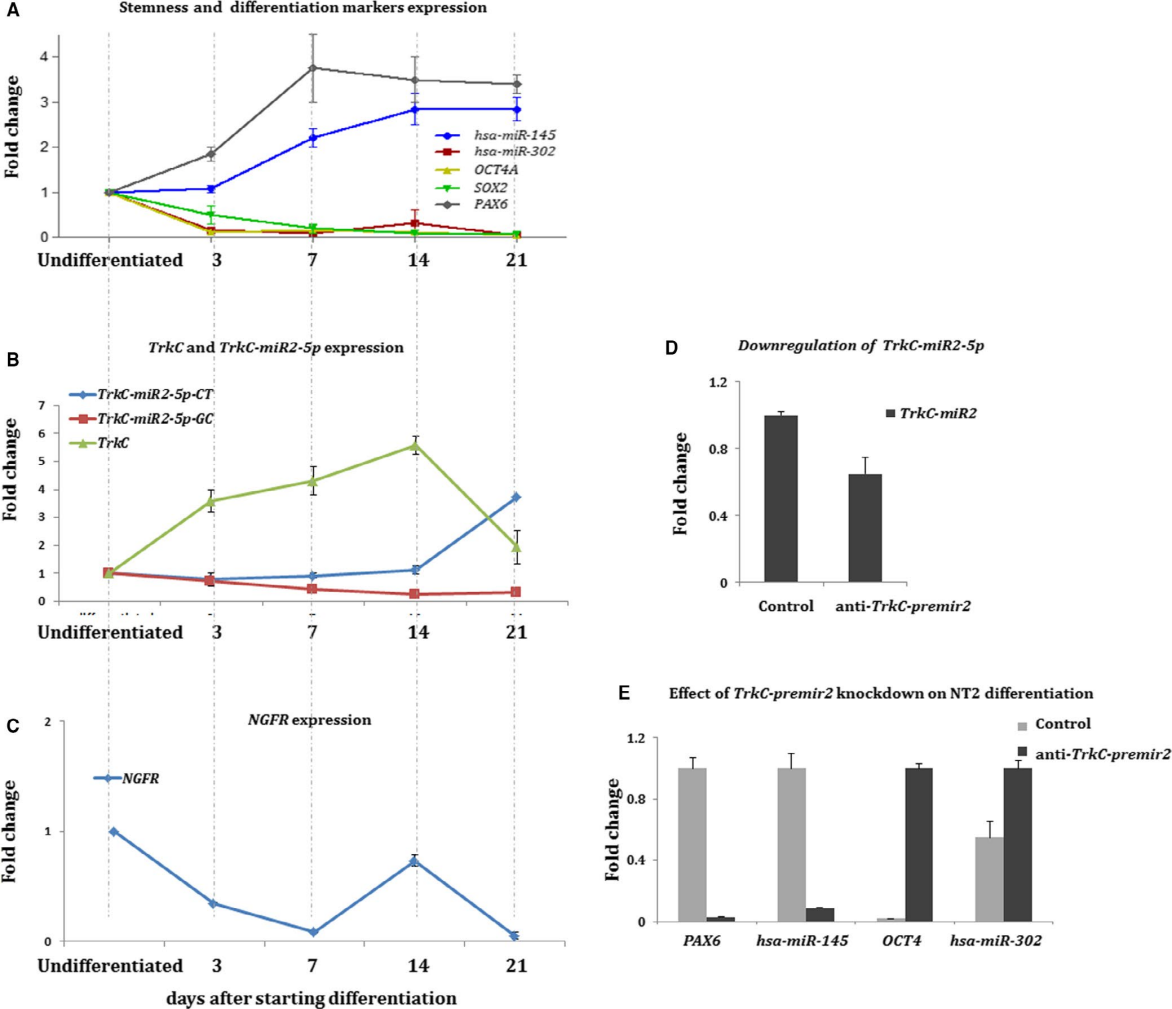
Expression profile of *TrkC* and *TrkC‐miR2* isomiRs for the duration of NT2 cell differentiation into neural‐like cells. A, The figure demonstrates *hsa‐miR‐145* up‐regulation and down‐regulation of *hsa‐miR‐302, Oct4* and *Sox2* markers for the period of NT2 differentiation, performed by RT‐qPCR. Data were compared with the undifferentiated cells. *GAPDH* was applied as an internal control for normalizing *Oct4* and *Sox2* expressions. *U48* expression was used for normalization of the expression of *hsa‐miR‐145* and *hsa‐miR‐302*. B, Tracking of the expression alteration of *TrkC‐miR2*‐5p isomiRs and *TrkC* throughout the differentiation of NT2 cells, detected by RT‐qPCR. As shown in this section, *TrkC‐miR2‐5p‐CT* is expressed in a reverse correlation with *TrkC* since 14th day of the course. C, A reverse correlation of expression was also deduced between *NGFR* gene and *TrkC‐miR2*‐5p‐CT since day 14th of the differentiation course. D, Shows successful down‐regulation of *TrkC‐miR2* following the application of an antisense sequence against *TrkC‐premir2* in NT2 cells. E, Down‐regulation of differentiation markers (*hsa‐miR‐145* and *PAX6*) and up‐regulation of stemness markers (*hsa‐miR‐302* and *Oct4A*) following the *TrkC‐premir2* knockdown by a construct containing its antisense sequence. All data were compared with the expression levels in the undifferentiated NT2 cells. *GAPDH* was applied for normalization of *PAX6* and *Oct4A U48* was employed as the internal control for normalization of the expression of *hsa‐miR‐145* and *hsa‐miR‐302*. Error bars indicate SD of duplicate experiments

As *TrkC* is primarily expressed in neural cells, the expression status of *TrkC‐miR2* was explored for the period of NT2 cell differentiation towards the neural‐like cells (Figure 5). This process was successfully accomplished in three weeks, and then *Sox2*, *Oct4A*, *hsa‐miR‐145*, *hsa‐miR‐302*, *PAX6*, *TrkC*, *TrkC‐miR2* and *NGFR* gene expression levels were weekly investigated. The expression of *Sox2*, *Oct4A* and *hsa‐miR‐302,* as the pluripotent markers, was significantly declined throughout the NT2 differentiation progress (Figure 5A). Unlike *TrkC‐miR2*‐5p‐GC, a major *TrkC‐miR2*‐5p‐CT expression elevation was noticed at the second week of NT2 differentiation course, which was coincident with notable *TrkC* expression decline (Figure 5B). Consistently, the expression of *NGFR* was reduced at the time that the expression of *TrkC‐miR2*‐5p‐CT was increased (Figure 5C).

*TrkC‐miR2* down‐regulation effect against the neural cell‐like differentiation was also investigated. To this aim, NT2 cells were first treated with RA (retinoic acid) in order to convince the cell differentiation and then were transfected with the vector containing anti‐*TrkC‐premir2,* 10 days after beginning of differentiation induction. Real‐time PCR results revealed a significant reduction (about 35% ) in *TrkC‐miR2* expression in these cells compared with the NT2 cells only treated with RA, as a control (Figure 5D). Following *TrkC‐miR2* suppression via anti‐*TrkC‐premir2*, *PAX6* and *hsa‐miR‐145* differentiation markers were significantly down‐regulated, whereas *OCT4A* and *hsa‐miR‐302* pluripotent markers were up‐regulated, 21 days after starting differentiation (Figure 5E).

## 4 DISCUSSION

MiRNAs are small non‐coding RNAs regulating many important cell processes such as differentiation via translation inhibition or mRNA degradation.^18^ It has been reported that *TrkC* receptor is implicated in neurotrophin signalling associated with various functions such as differentiation, cell death and cell survival.^19^, ^20^ On the other hand, the mechanism(s) explaining the *TrkC* contradictory functions is/are not well known yet. Hence, finding novel factors regulating neurotrophin signalling pathway may make available the cell fate manipulation opportunity in some diseases such as cancers and tissue regeneration. Our prior attempt led to the prediction of a unique *bona fide* stem loop structure named *TrkC‐premir2* using multiple software (SSC profiler, miPRED, CID‐miRNA software along with Microprocessor SVM program, Mireval, MatureBayes, Pmirp, miRNA Spotter, MiRmat, and MirZ, and RNAfold algorithm) and discovery of a novel miRNA, named *TrkC‐miR2*, which is located in *TrkC* gene and implicated in Wnt signalling pathway regulation.^11^ Also, in our previous research, following the overexpression of *TrkC‐miR2* precursor, we tested the production of both predicted *TrkC‐miR2*‐5p and *TrkC‐miR2*‐3p levels using specific RT‐qPCR. However, only *TrkC‐miR2*‐5P was amplifiable, probably due to the more stability of it. Consistently, the number of reads for *TrkC‐miR2*‐3p sequence in the RNAseq data was much lower than *TrkC‐miR2*‐5p.^11^ Here, we presented in silico study and supportive experimental evidence revealing *TrkC‐miR2* has the potential to be under consideration as a main regulator implicated in neurotrophin signalling pathway.

### 4.1 Association between neurotrophin signalling pathway and *TrkC‐miR2*

MiRNAs, as the key regulatory factors in the cells, apply their effects via target mRNAs degradation or their translation inhibition.^21^ RT‐qPCR data revealed that *NGFR* (a key gene in neurotrophin signalling pathway) is down‐regulated following the overexpression of *TrkC‐premir2* (Figure 1B), which was then verified by performing Western blotting against NGFR (Figure 1C). Furthermore, direct interaction of *NGFR* 3'UTR with *TrkC‐miR2* was supported by dual‐luciferase assay (Figure 1D).

*NGFR*, *TrkA*, *TrkB* and *TrkC* are categorized as the neurotrophin receptors, which function alone or in cooperation with each other.^1^ *TrkA* with the help of its downstream gene, *Akt,* leads the cell to survival fate, whereas *NGFR* is a cell surface receptor with multi‐functional role, which is implicated in injury, regeneration and development of nervous system, and also acts as a tumour suppressor.^8^, ^22^ *BCl2* is an anti‐apoptotic gene working downstream of *NF‐kB*, which in turn results in survival of the cell.^23^ Overexpression of *TrkC‐miR2* resulted in *NGFR* down‐regulation (Figure 1) and up‐regulation of *TrkA*, *Akt* and *BCl2* (Figure 2), which is expected to result in more survival of transfected cells. Up‐regulation of neurotrophin downstream genes is consistent with increased survival effect, which is shown by flow cytometry and MTT assay results against the cells overexpressing *TrkC‐miR2* (Figure 4). The result is consistent with the previously described survival influence of *TrkC*,^24^, ^25^ which emphasizes on the cellular functionality of *TrkC‐miR2* corresponding to the function of its host gene, *TrkC*. Interestingly, *NF‐κB* was up‐regulated (Figure 2) following *TrkC‐premir2* overexpression, whereas *NGFR* was down‐regulated (Figure 1). It suggests that *TrkC‐miR2* might be involved in neurotrophin signalling pathway in an *NGFR*‐independent pathway.^26^

### 4.2 Uncovering of *TrkC‐miR2* expression in brain cell lines and tumour specimens

Both isomiRs of *TrkC‐miR2* were identified in several cancer tissues and cell lines (Figure 3A, B, C), similar to the *TrkC* as the host gene of this miRNA.^27^, ^28^, ^29^ The comparative higher expression level of this miRNA in glioblastoma samples and cell lines may identify this miRNA candidate as a glioma biomarker. On the other hand, opposite expression level of *TrkC‐miR2‐5p‐GC* related to the *NGFR* and *TrkC* genes in tumour tissue samples supports the functionality of this miRNA against *NGFR* and *TrkC* (Figure 3B,C).

### 4.3 Induction of cell survival through ectopic expression of *TrkC‐premir2*

Flow cytometry, annexin V test and also MTT assay in U87 and HEK293 t cells transfected with a construct overexpressing *TrkC‐premir2* showed significant increase in the rate of cell survival (Figure 4). These results were consistent with the survival effect of *TrkC* gene, which has been previously described ^25^ that emphasizes the effectiveness of *TrkC‐miR2* along with its host gene, *TrkC*. U87 cell line expresses the genes that are implicated in neurotrophin signalling pathway actively.^30^ Up‐regulation of neurotrophin signalling pathway genes following *TrkC‐miR2* overexpression (Figure 2) is consistent with its survival effect in U87 cells overexpressing *TrkC‐premir2* (Figure 4). This is also consistent with the effect of *TrkC‐premir2* overexpression in SW480 cell line in which Wnt signalling pathway is prominent.^11^

### 4.4 *TrkC‐miR2* expression is altered in the course of NT2 differentiation into neural‐like cells

As *Trk* genes are identified to be implicated in neural cell differentiation ^1^, ^31^, ^32^, ^33^ and also *TrkC‐miR2* is significantly expressed in glioma‐originated cancers and cell lines (Figure 3) and targets *NGFR* (Figure 1), the expression effect of *TrkC‐miR2* was investigated for the duration of NT2 cell differentiation into neural‐like cells ^17^ (Figure 5). Results showed that *TrkC‐miR2* expression alteration was in reverse correlation with *NGFR* expression particularly since day 14 of the differentiation (Figure 5C). Down‐regulation of *TrkC‐miR2* (Figure 5D) attenuated differentiation outcome (Figure 5E) supporting the fundamental role of this miRNA during the differentiation of NT2 cells possibly through targeting of *NGFR*.^19^, ^20^, ^31^, ^32^ It remained to be examined whether further *TrkC‐miR2*‐predicted target genes are expressed in opposite association.

## 5 CONCLUSION

In conclusion, here we introduced accumulative evidence showing the function of *TrkC‐miR2* against the components of neurotrophin signalling pathway. Altogether, the presented evidence identifies this miRNA candidate as a controller of neurotrophin pathway and its implication in differentiation of neural cells.

## CONFLICT OF INTERESTS

The authors declare that there is no conflict of interest with any financial organization regarding the material discussed in the manuscript.

## AUTHOR CONTRIBUTION

**Sadat Dokaneheifard:** Data curation (equal); Formal analysis (equal); Investigation (equal); Methodology (equal); Project administration (equal); Software (equal); Validation (equal); Writing‐original draft (equal); Writing‐review & editing (equal). **Bahram Mohammad Soltani:** Conceptualization (equal); Data curation (equal); Formal analysis (equal); Funding acquisition (equal); Methodology (equal); Project administration (equal); Resources (equal); Supervision (equal); Validation (equal); Visualization (equal); Writing‐review & editing (equal).

## ETHICAL APPROVAL

Tissue samples were obtained from Imam Hospitals, Tehran/Iran. All these samples have been used with getting satisfying with all donors. The Tarbiat Modares University Ethics Committee approved the study.

## Data Availability

The data sets used and/or analysed during the current study are available from the corresponding author on reasonable request.

## References

[1] Reichardt LF . Neurotrophin‐regulated signalling pathways. Philos Trans R Soc Lond B Biol Sci. 2006;361:1545‐1564. 16939974 10.1098/rstb.2006.1894 PMC1664664

[2] McGregor LM , McCune BK , Graff JR , et al. Roles of trk family neurotrophin receptors in medullary thyroid carcinoma development and progression. Proc Natl Acad Sci. 1999;96:4540‐4545. 10200298 10.1073/pnas.96.8.4540 PMC16368

[3] Genevois A‐L , Ichim G , Coissieux M‐M , et al. Dependence receptor TrkC is a putative colon cancer tumor suppressor. Proc Natl Acad Sci. 2013;110:3017‐3022. 23341610 10.1073/pnas.1212333110 PMC3581924

[4] Jin W , Kim GM , Kim MS , et al. TrkC plays an essential role in breast tumor growth and metastasis. Carcinogenesis. 2010;31:1939‐1947. 20802235 10.1093/carcin/bgq180

[5] Luo Y , Kaz AM , Kanngurn S , et al. NTRK3 is a potential tumor suppressor gene commonly inactivated by epigenetic mechanisms in colorectal cancer. PLoS Genet. 2013;9:e1003552. 23874207 10.1371/journal.pgen.1003552 PMC3708790

[6] Tsang JY , Wong KH , Lai MW , et al. Nerve growth factor receptor (NGFR): a potential marker for specific molecular subtypes of breast cancer. J Clin Pathol. 2012;66(4):291‐296. 23268325 10.1136/jclinpath-2012-201027

[7] Aranha MM , Santos DM , Solá S , Steer CJ , Rodrigues C . miR‐34a regulates mouse neural stem cell differentiation. PLoS One. 2011;6:e21396. 21857907 10.1371/journal.pone.0021396 PMC3153928

[8] Parsi S , Soltani BM , Hosseini E , Tousi SE , Mowla SJ . Experimental verification of a predicted intronic microRNA in human NGFR gene with a potential pro‐apoptotic function. PLoS One. 2012;7:e35561. 22558167 10.1371/journal.pone.0035561 PMC3338703

[9] Hassanlou M , Soltani BM , Mowla SJ . Expression and function of hsa‐miR‐6165 in human cell lines and during the NT2 cell neural differentiation process. J Mol Neurosci. 2017;63:254‐266. 28956260 10.1007/s12031-017-0954-5

[10] Dokanehiifard S , Soltani BM , Parsi S , Hosseini F , Javan M , Mowla SJ . Experimental verification of a conserved intronic microRNA located in the human TrkC gene with a cell type‐dependent apoptotic function. Cell Mol Life Sci. 2015;72(13):2613–2625. 25772499 10.1007/s00018-015-1868-4 PMC11113298

[11] Dokanehiifard S , Yasari A , Najafi H , et al. A novel microRNA located in TrkC gene regulates Wnt signaling pathway and is differentially expressed in colorectal cancer specimens. J biol chem. 2017;292(18):7566‐7577. 28100780 10.1074/jbc.M116.760710 PMC5418054

[12] Dokanehiifard S , Soltani BM . TrkC‐miR2 regulates TGFβ signaling pathway through targeting of SMAD3 transcript. J Cell Biochem. 2019;120:2634‐2641. 10.1002/jcb.27572 30304551

[13] Sambrook J , Fritsch E , Maniatis T . Molecular cloning: A laboratory manual+ Cold Spring Harbor. 1989. Cold Spring Harbor Laboratory Press, New York.

[14] Xu N , Papagiannakopoulos T , Pan G , Thomson JA , Kosik KS . MicroRNA‐145 regulates OCT4, SOX2, and KLF4 and represses pluripotency in human embryonic stem cells. Cell. 2009;137:647‐658. 19409607 10.1016/j.cell.2009.02.038

[15] Dokanehiifard S , Soltani BM . Hsa‐miR‐11181 regulates Wnt signaling pathway through targeting of APC2 transcripts in SW480 cell line. Gene. 2018;641:297‐302. 29111205 10.1016/j.gene.2017.10.075

[16] Livak KJ , Schmittgen TD . Analysis of relative gene expression data using real‐time quantitative PCR and the 2(‐Delta Delta C(T)) Method. Methods (San Diego, Calif). 2001;25:402‐408. 10.1006/meth.2001.1262 11846609

[17] Andrews PW . Retinoic acid induces neuronal differentiation of a cloned human embryonal carcinoma cell line in vitro. Dev Biol. 1984;103:285‐293. 6144603 10.1016/0012-1606(84)90316-6

[18] Dalmay T . MicroRNAs and cancer. J Intern Med. 2008;263:366‐375. 18298485 10.1111/j.1365-2796.2008.01926.x

[19] Hapner SJ , Boeshore KL , Large TH , Lefcort F . Neural differentiation promoted by truncated trkC receptors in collaboration with p75 NTR. Dev Biol. 1998;201:90‐100. 9733576 10.1006/dbio.1998.8970

[20] Verdi JM , Birren SJ , Ibáñez CF , et al. p75 LNGFR regulates Trk signal transduction and NGF‐induced neuronal differentiation in MAH cells. Neuron. 1994;12:733‐745. 7512816 10.1016/0896-6273(94)90327-1

[21] Krol J , Loedige I , Filipowicz W . The widespread regulation of microRNA biogenesis, function and decay. Nat Rev Genet. 2010;11:597. 20661255 10.1038/nrg2843

[22] Johnston A , Lun X , Rahn JJ , et al. The p75 neurotrophin receptor is a central regulator of glioma invasion. PLoS Biol. 2007;5:e212. 17696644 10.1371/journal.pbio.0050212 PMC1939884

[23] Busca A , Saxena M , Kryworuchko M , Kumar A . Anti‐apoptotic genes in the survival of monocytic cells during infection. Curr Genomics. 2009;10:306. 20119528 10.2174/138920209788920967 PMC2729995

[24] Kumar S , Kahn MA , Dinh L , de Vellis J . NT‐3‐mediated TrkC receptor activation promotes proliferation and cell survival of rodent progenitor oligodendrocyte cells in vitro and in vivo. J Neurosci Res. 1998;54:754‐765. 9856859 10.1002/(SICI)1097-4547(19981215)54:6<754::AID-JNR3>3.0.CO;2-K

[25] Minichiello L , Klein R . TrkB and TrkC neurotrophin receptors cooperate in promoting survival of hippocampal and cerebellar granule neurons. Genes Dev. 1996;10:2849‐2858. 8918886 10.1101/gad.10.22.2849

[26] Prencipe G , Minnone G , Strippoli R , et al. Nerve growth factor downregulates inflammatory response in human monocytes through TrkA. J Immunol. 2014;192:3345‐3354. 24585880 10.4049/jimmunol.1300825

[27] Brodeur GM , Nakagawara A , Yamashiro DJ , et al. Expression of TrkA, TrkB and TrkC in human neuroblastomas. J Neurooncol. 1997;31:49‐56. 9049830 10.1023/a:1005729329526

[28] Lomen‐Hoerth C , Shooter EM . Widespread neurotrophin receptor expression in the immune system and other nonneuronal rat tissues. J Neurochem. 1995;64:1780‐1789. 7891106 10.1046/j.1471-4159.1995.64041780.x

[29] Yamamoto M , Sobue G , Yamamoto K , Mitsuma T . Expression of mRNAs for neurotrophic factors (NGF, BDNF, NT‐3, and GDNF) and their receptors (p75 NGFR, TrkA, TrkB, and TrkC) in the adult human peripheral nervous system and nonneural tissues. Neurochem Res. 1996;21:929‐938. 8895847 10.1007/BF02532343

[30] Lu D‐Y , Leung Y‐M , Cheung C‐W , Chen Y‐R , Wong K‐L . Glial cell line‐derived neurotrophic factor induces cell migration and matrix metalloproteinase‐13 expression in glioma cells. Biochem Pharmacol. 2010;80:1201‐1209. 20615395 10.1016/j.bcp.2010.06.046

[31] Calvo L , Anta B , López‐Benito S , et al. Bex3 dimerization regulates NGF‐dependent neuronal survival and differentiation by enhancing trkA gene transcription. J Neurosci. 2015;35:7190‐7202. 25948268 10.1523/JNEUROSCI.4646-14.2015 PMC6605261

[32] Diolaiti D , Bernardoni R , Trazzi S , et al. Functional cooperation between TrkA and p75 NTR accelerates neuronal differentiation by increased transcription of GAP‐43 and p21 (CIP/WAF) genes via ERK1/2 and AP‐1 activities. Exp Cell Res. 2007;313:2980‐2992. 17619016 10.1016/j.yexcr.2007.06.002

[33] Nogueira E , Navarro S , Pellín A , Llombart‐Bosch A . Activation of TRK genes in Ewing's Sarcoma Trk a receptor expression linked to neural differentiation. Diagn Mol Pathol. 1997;6:10‐16. 9028732 10.1097/00019606-199702000-00003

